# Upper beak depression instead of elevation dominates cranial kinesis in woodpeckers

**DOI:** 10.1098/rsbl.2023.0148

**Published:** 2023-06-07

**Authors:** S. Lyons, S. Baeckens, S. Van Wassenbergh

**Affiliations:** ^1^ Laboratorio de Anatomía Comparada, Facultad de Ciencias Naturales y Museo, Universidad Nacional de La Plata, 1900 La Plata, Buenos Aires, Argentina; ^2^ Evolution and Optics of Nanostructures Lab, Department of Biology, Ghent University, 9000 Gent, Belgium; ^3^ Laboratory of Functional Morphology, Department of Biology, University of Antwerp, 2610 Antwerpen, Belgium

**Keywords:** birds, cranial kinesis, biomechanics, functional morphology, ecomorphology, jaws

## Abstract

The value of birds’ ability to move the upper beak relative to the braincase has been shown in vital tasks like feeding and singing. In woodpeckers, such cranial kinesis has been thought to hinder pecking as delivering forceful blows calls for a head functioning as a rigid unit. Here, we tested whether cranial kinesis is constrained in woodpeckers by comparing upper beak rotation during their daily activities such as food handling, calling and gaping with those from closely related species that also have a largely insectivorous diet but do not peck at wood. Both woodpeckers and non-woodpecker insectivores displayed upper beak rotations of up to 8 degrees. However, the direction of upper beak rotation differed significantly between the two groups, with woodpeckers displaying primarily depressions and non-woodpeckers displaying elevations. The divergent upper beak rotation of woodpeckers may be caused either by anatomical modifications to the craniofacial hinge that reduce elevation, by the caudal orientation of the mandible depressor muscle forcing beak depressions, or by both. Our results suggest that pecking does not result in plain rigidification at the upper beak's basis of woodpeckers, but it nevertheless significantly influences the way cranial kinesis is manifested.

## 1. Introduction

Cranial kinesis is present in most neognathous birds [1,2]. It comes in a variety of forms, but prokinesis or the rotation of the maxillary rostrum (i.e. the upper beak/jaw) as a rigid unit about the nasofrontal hinge is the most common type [1,3–5]. A prokinetic elevation of the upper beak is classically described to occur when the mandible (i.e. the lower beak/jaw) depresses, and hence the two beak halves open or close in synchrony [1,6,7]. However, later studies showed an important level of independence in the control of upper and lower beak movement [3,8–11].

While the adaptive advantage over akinetic skulls (i.e. skulls with a fixed upper beak) is not always clear, selective forces promoting cranial kinesis strongly depend on the ecological context [3]. Roles of cranial kinesis have been hypothesized in improving motion dynamics and control of food handling [1,11–14], in sound production during singing [9] and in preventing injuries by shock absorption [1,15]. Mechanical trade-offs with other beak functions, however, cannot be excluded. For example, mathematical model calculations predict a reduced biting performance in kinetic skulls compared with akinetic skulls [3].

Cranial kinesis of woodpeckers (Picidae) is hypothesized to be selected against because of its counterproductive effect on forceful pecking [16,17]. According to Bock [16], the elevation of the upper beak is inhibited during the beak's pecking impact because of the frontal overhang—a thickening of the frontal bone that bulges over the most caudal part of the upper beak, thereby potentially blocking the dorsal rotation of the upper beak. Frontal overhang evolved in the ancestral lineage of piculets (Picumninae) and true woodpeckers (Picinae *s.l.*), but became secondarily reduced in the most derived clade, the Malarpicini [17–22]. During the impact phase of pecking, woodpecker heads were observed to behave remarkably stiffly: neither elevations nor depressions of the upper beak were detected [23]. This seems to confirm that adaptations to restrict cranial kinesis are at play. However, after the phase of impact, significant cranial kinesis does occur during retraction of the beak from the tree, involving both upper beak elevation and depression [24].

Here, we tested the hypothesis that pecking is linked with an overall constrained cranial kinesis (i.e. magnitude of either upper beak elevation or depression), and with a restricted upper beak elevation in particular [16], by comparing cranial kinesis during diverse activities such as singing, food handling, drinking and gaping in woodpeckers with cranial kinesis in closely related bird species with a similar diet that do not use their beaks to hit trees.

## 2. Methods

### (a) Video analysis

**Figure 1.**
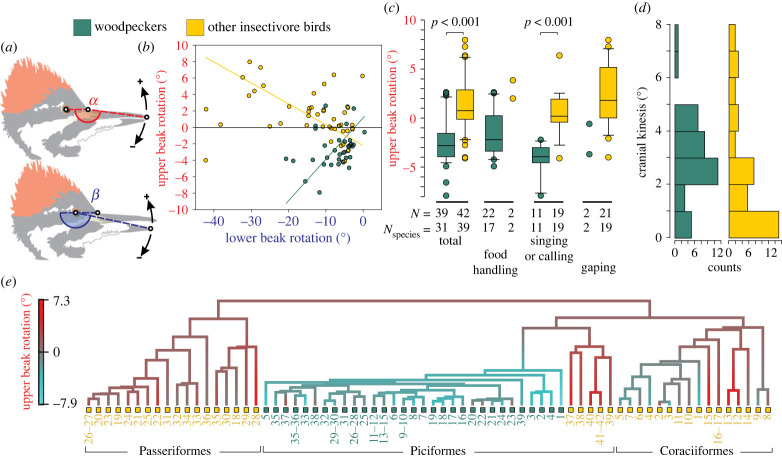
Comparative data on cranial kinesis in woodpeckers (green points or bars) versus other insectivore birds (yellow points or bars). (*a*) Illustration of upper beak angle *α* and lower beak angle *β*, defining rotation directions. (*b*) Linear regression results of *α* versus *β* rotations showing an inverse relationship in the two groups. (*c*) Box-and-whisker plots (central bar = median; box boundaries = 25th and 75th percentiles; whiskers = 10th and 90th percentiles; circles = outliers) of upper beak rotation in woodpeckers (green) and closely related insectivores (yellow) for the pooled data (total) and split up in behavioural categories. Statistical test probabilities are given at the top. In (*d*), histograms of absolute values of upper beak rotation display the distribution of cranial kinesis magnitude. (*e*) Phylogeny of the 70 species (numbers defined in electronic supplementary material, tables S1 and S2) showing ancestral state estimation [26] of upper beak rotation along the branches (high negative values in cyan; high positive values in red).

About 10 000 video clips from the Macaulay Library of wildlife recordings of Cornell University [25] were screened. Video fragments of beak movements from an approximately lateral perspective (deviations less than 20° in yaw, and less than 10° in roll; implying less than 12% error in measured beak rotations—electronic supplementary material, figure S1) were selected. Two video frames were extracted for each movement sequence: a first frame with the beak closed just before the start of beak opening, and a second frame with the beak at maximum gape. The upper beak angle *α* between three landmarks (figure 1*a*) was measured on each image using TpsDig software [27]: (1) the centre of the eye, (2) the approximate location of the nasofrontal joint, and (3) the upper beak tip. Lower beak angle *β* was estimated using the abovementioned landmarks (1) and (2), and (3) the lower beak tip (figure 1*a*). To allow subpixel coordinate digitizations, the pixel resolution of the images was increased by 2.5 times in both height and width using Adobe Photoshop (Adobe Systems, San Jose, USA). Upper and lower beak rotations were calculated by subtracting the closed-beak *α* or *β* from, respectively, the *α* or *β* at maximum gape. Our angle definition (figure 1*a*) implies that positive values denote beak elevations with respect to the cranium. The total amount of cranial kinesis irrespective of the direction of the rotation was obtained by taking the absolute value of upper beak rotation. To account for error in landmark placement, angle measurements were repeated three times and the averages were used in the analyses.

Beak rotations were measured from 39 video clips of woodpeckers (electronic supplementary material, file S1) and from 41 videos of other families of insectivorous birds from their two most closely related orders (i.e. Coraciiformes and Passeriformes [28]) and from the order Piciformes (electronic supplementary material, file S2). As one video of an insectivorous bird contained two beak movement sequences, 42 beak sequences were analysed for the insectivorous birds. A total of 70 species were studied: 31 woodpecker species belonging to the family Picidae and 39 closely related insectivorous species from 18 different families. Diet preference for insects was determined based on the literature [29,30]. The activities of the birds in each video were assigned to four behavioural categories: food handling (including beak movement after swallowing or drinking the sap of a tree), singing/calling, gaping (i.e. perched beak opening without apparent ecological function) and drinking water (only observed in woodpeckers). The number of observations per category and per bird group is reported in figure 1*c*. Osteological nomenclature was taken from Baumel *et al*. [31].

### (b) Statistical analysis

Differences in upper beak rotation between pecking and non-pecking species were assessed using traditional (*t*-test) and phylogenetic statistics. Parametric *t*-tests were possible since data distributions for upper beak angles did not differ from normality in the groups (Shapiro–Wilk test), and no significant differences in variance were found (two-sample *F*-test for variance). Phylogenetic analyses were run on the complete Bayesian maximum clade credibility species-level avian phylogeny [32] pruned to the 70 species used in this study. If species were not represented in the tree, the phylogenetic position of closely related congeneric species was used (see electronic supplementary material, files S1 and S2). A phylogenetic ANOVA (‘phytools’ package [33]) tested for a difference in total upper beak rotation between pecking and non-pecking species while controlling for phylogenetic non-independence. Phylogenetic signal strength of woodpecking behaviour was calculated using Fritz & Purvis's *D*-test for binary traits [34]. Lastly, reduced major axis regressions were used to determine the relationships between upper and lower beak rotation within these two groups of birds using the pooled dataset of different behaviours. Ninety-five per cent confidence intervals were determined by bootstrapping with 1999 replicates using Past 4.04 (Øyvind Hammer, University of Oslo).

## 3. Results

When pooling the measurements from different behaviours, upper beak rotations in woodpeckers ranged from depressions of −7.91 degrees (*Jynx torquilla*; singing/calling) to elevations of 2.63 (*Melanerpes formicivorus*; food handling) (figure 1*b*; electronic supplementary material, file S1). Depressions of the upper beak during lowering of the mandible were observed in 13 of the 14 woodpecker genera studied. In our sample of other insectivore birds, the upper beak rotated between −4.1 degrees (*Alcedo atthis*: Alcedinidae; singing) and 7.97 degrees (*Galbula cyanescens*: Galbulidae; gaping) with respect to the cranium (electronic supplementary material, file S2). Among the non-woodpecker insectivores, birds from three coraciiform families (Alcedinidae, Cerylidae and Coraciidae) showed depression of the upper beak, representing 15% of the species analysed. Neither Passeriformes nor the other families of Coraciiformes and non-pecking Piciformes analysed presented depression of the upper beak. In *Schoeniophylax phryganophilus* (Furnariidae), the elevation of the upper beak without movement of the lower beak was observed (6.25 degrees of upper beak rotation), but in the same video (same individual) also a beak opening was observed with depression of the mandible showing virtually no movement (−0.18 degrees rotation) of the upper beak.

Increasing cranial kinesis was observed for increasing lower beak depressions (figure 1*b*). The slope of upper versus lower beak rotation showed an opposite relationship in woodpeckers compared with the other insectivore birds: increasing lower beak depressions were related to increasing *depressions* of the upper beak in woodpeckers (95% confidence of slope *α*/*β* = 0.29 to 1.54, *p* < 0.0001; *r* = 0.26), and to increasing *elevations* of the upper beak in the other insectivore birds (95% confidence of slope *α*/*β* = −0.75 to −0.18, *p* < 0.0001; *r* = −0.29) (figure 1*b*).

Mean upper beak rotations in woodpeckers and other insectivore birds were, respectively, −2.7 ± 2.1 degrees and 1.3 ± 2.7 degrees (mean ± s.d.), and differed significantly between two groups (*t* = −6.77, d.f. = 68, *p* < 0.001, two-tailed) (figure 1*c*). Similar differences were present in upper beak rotation during singing or calling, with respectively −4.3 ± 1.6 and 0.36 ± 2.29 degrees (*t* = −5.97, d.f. = 28, *p* < 0.001, two-tailed). The same trend in upper beak rotations was observed within food handling and gaping, but no statistical tests were possible owing to the limited number of samples for these behaviours (figure 1*c*). Cases with limited cranial kinesis (less than 1 degree) appeared less frequent in woodpeckers (12.8%) than in the other insectivores (35.7%) when considering all behaviours pooled together (figure 1*d*). The mean and median values of cranial kinesis were higher in woodpeckers (2.51 and 2.8 degrees) than in the other insectivore birds (1.48 and 0.74 degrees) (figure 1*d*).

Contrary to traditional statistics, phylogenetically informed statistics did not reveal a significant difference in upper beak rotation between woodpeckers and non-woodpecking insectivore birds (*F* = 45.8; *p* = 0.198) (figure 1*e*). This result was expected because woodpecking displays an extremely clumped phylogenetic pattern (*D* = −1.21, *p* < 0.001) as the trait evolved only once in the woodpecker lineage of Picidae (figure 1*e*).

## 4. Discussion

Our findings do not support the hypothesis that overall cranial kinesis (regardless of upper beak rotation direction) is reduced in woodpeckers as a likely adaptation to forceful pecking (figure 1*d*). However, the hypothesis that elevation of the upper beak is generally restricted in woodpeckers was confirmed. A depression of the upper beak in woodpeckers was generally observed, while in closely related insectivores the upper beak typically is elevated (figure 1*b,c,e*). Although phylogenetically informed statistics could not prove that this pattern of upper beak movement is an adaptation to pecking at wood in the analysed taxa, the power of this test was inevitably limited owing to the woodpeckers’ monophyly (figure 1*e*). In line with previous measurements of beak kinematics during beak retraction after pecking [24], upper beak elevation was still observed regularly in our sample of woodpeckers, but never higher than 2.6 degrees. The other insectivores occasionally showed elevations higher than 5 degrees (figure 1*b–d*). Further work may test whether the variation in the upper beak's range of motion between species can be linked to the morphology of the nasofrontal joint, such as the frontal overhang.

**Figure 2.**
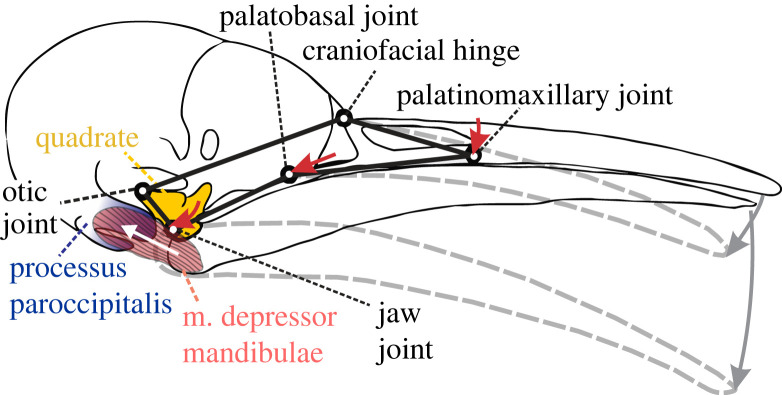
Upper beak depression linked with quadrate retraction transmitted through the pterygoid–palatine complex in a woodpecker skull. The beak is shown at rest (full lines) and after opening involving upper beak depression (grey dashed lines). A five-bar linkage illustrates the coupled effects of posterior movement of the jaw joint (red arrows). The m. depressor mandibulae's origin on the processus paroccipitalis (blue) implies a caudodorsally directed line of action (white arrow), which may drive upper beak depression during forceful beak opening. An animation is included as electronic supplementary material, video S1.

As the abovementioned frontal overhang could restrict elevation but will not generate upper beak depressions, what explains this movement in woodpeckers? The line of action of the m. depressor mandibulae may play a role [35]. Previous studies pointed out that contraction of a forward inclined (from insertion on the mandible to a more rostrodorsal origin) depressor muscle causes the quadrate to rotate counterclockwise (when the bird is viewed with the beak pointing to the right) and pushes the upper beak up via rostral movement of the pterygoid–palatine complex as well as the jugal bone [5]. However, the m. depressor mandibulae's line of action in woodpeckers is strongly rearward (i.e. from insertion on the mandible to a more caudodorsal origin) [20], even almost parallel to the mandible, as a consequence of the cranioventral development of the processus paroccipitalis (rostral process at caudolateral margin of meatus acusticus *sensu* [22]), onto which the depressor mandibulae inserts in Picinae (figure 2). As a result, force from the m. depressor mandibulae of woodpeckers can cause rotation of the quadrate in the opposite direction, which would depress the upper beak at the same time as the lower beak is depressed (see electronic supplementary material, video S1). The commonly observed depression of the upper beak in woodpeckers could thus be related to their specific musculoskeletal morphology of the otic region.

Our data show that coupled kinesis where the maxilla elevates as the mandible depresses is generally absent in woodpeckers (figure 1*b*), despite their generally having the postorbital ligament [20], which has been shown to produce this coupled movement [1]. Also for the other insectivorous birds, we found that the upper beak can be raised or remain immobile as the mandible depresses during different activities. Even elevation and absence of movement can occur in the same species, and elevation of the upper beak was observed without lowering of the mandible. Such upper beak elevations while the mandible remains static were previously considered rare, with records for only the Charadriformes of the genus *Scolopax* [1] and the species *Rhynchops niger* [16], and for the columbiform species *Columba livia* [8]. Our measurements are, to our knowledge, the first observation for Passeriformes (*S. phryganophilus*). This movement may thus be more common than originally thought. Together, these findings suggest that birds have an elaborate control of upper beak movement by the musculature of the quadrates and pterygoid bones. This is in line with several studies showing that a stringent role of the postorbital ligament in upper beak elevation coupled to mandible depression is often absent [9,10,35].

In conclusion, our study showed that cranial kinesis is not reduced in woodpeckers, but instead shifted predominantly towards depressions instead of elevations of the upper beak. This may be linked to presumed morphological adaptions to pecking, such as a frontal overhang, resisting strong upper beak elevation as hypothesized earlier [16]. We predict that the caudally inclined line of action of the lower beak depressor muscles contributes to generating this type of cranial kinesis (figure 2). Evidence was found for control of cranial kinesis independent of lower beak movement within both woodpeckers and closely related insectivore species. To unravel the adaptive value of skulls with depression-dominated cranial kinesis for pecking, and to better understand the potential trade-offs with performance or efficiency of actions such as food handling, drinking or preening, further biomechanical work will be needed.

## Data Availability

Online video resource locators and raw data are provided in the electronic supplementary material [36].
